# Ecological Niche Modelling Predicts Southward Expansion of *Lutzomyia (Nyssomyia) flaviscutellata* (Diptera: Psychodidae: Phlebotominae), Vector of *Leishmania (Leishmania) amazonensis* in South America, under Climate Change

**DOI:** 10.1371/journal.pone.0143282

**Published:** 2015-11-30

**Authors:** Bruno M. Carvalho, Elizabeth F. Rangel, Paul D. Ready, Mariana M. Vale

**Affiliations:** 1 Laboratório de Vertebrados, Instituto de Biologia, Universidade Federal do Rio de Janeiro, Rio de Janeiro, Brazil; 2 Laboratório Interdisciplinar de Vigilância Entomológica em Diptera e Hemiptera, Instituto Oswaldo Cruz, Fundação Oswaldo Cruz, Rio de Janeiro, Brazil; 3 Department of Disease Control, Faculty of Infectious and Tropical Diseases, London School of Hygiene and Tropical Medicine, London, United Kingdom; 4 Pós-Graduação em Ecologia e Evolução, Universidade do Estado do Rio de Janeiro, Rio de Janeiro, Brazil; University of Queensland & CSIRO Biosecurity Flagship, AUSTRALIA

## Abstract

Vector borne diseases are susceptible to climate change because distributions and densities of many vectors are climate driven. The Amazon region is endemic for cutaneous leishmaniasis and is predicted to be severely impacted by climate change. Recent records suggest that the distributions of *Lutzomyia (Nyssomyia) flaviscutellata* and the parasite it transmits, *Leishmania (Leishmania) amazonensis*, are expanding southward, possibly due to climate change, and sometimes associated with new human infection cases. We define the vector’s climatic niche and explore future projections under climate change scenarios. Vector occurrence records were compiled from the literature, museum collections and Brazilian Health Departments. Six bioclimatic variables were used as predictors in six ecological niche model algorithms (BIOCLIM, DOMAIN, MaxEnt, GARP, logistic regression and Random Forest). Projections for 2050 used 17 general circulation models in two greenhouse gas representative concentration pathways: “stabilization” and “high increase”. Ensemble models and consensus maps were produced by overlapping binary predictions. Final model outputs showed good performance and significance. The use of species absence data substantially improved model performance. Currently, *L*. *flaviscutellata* is widely distributed in the Amazon region, with records in the Atlantic Forest and savannah regions of Central Brazil. Future projections indicate expansion of the climatically suitable area for the vector in both scenarios, towards higher latitudes and elevations. *L*. *flaviscutellata* is likely to find increasingly suitable conditions for its expansion into areas where human population size and density are much larger than they are in its current locations. If environmental conditions change as predicted, the range of the vector is likely to expand to southeastern and central-southern Brazil, eastern Paraguay and further into the Amazonian areas of Bolivia, Peru, Ecuador, Colombia and Venezuela. These areas will only become endemic for *L*. *amazonensis*, however, if they have competent reservoir hosts and transmission dynamics matching those in the Amazon region.

## Introduction

The latest report of the Intergovernmental Panel on Climate Change (IPCC) states that climate change will affect human health through exacerbation of health problems that already exist [1,2]. Vector borne diseases are particularly susceptible to climate change because the distributions of the species involved in the complex transmission cycles are highly related to climatic variables. Under the assumption that species occupy only climatically suitable areas, changes in the geographical distribution of infectious diseases vectors are expected [3–5]. Leishmaniases are climate-sensitive diseases, not least because the distribution and behaviour of their sand fly vectors are strongly affected by rainfall, temperature and humidity [6,7]. The current report investigates the potential effects of climate change on the spatial distribution of *Lutzomyia (Nyssomyia) flaviscutellata* (Mangabeira) (Diptera, Psychodidae), a phlebotomine sand fly vector [8] of the parasitic protozoan *Leishmania (Leishmania) amazonensis* Lainson & Shaw (Kinetoplastida, Trypanosomatidae), a causative agent of zoonotic cutaneous leishmaniasis (ZCL) throughout much of tropical South America [9,10].

Leishmaniases are among the world’s six most neglected diseases, affecting men, women and children. The World Health Organization estimates the yearly occurrence as about 200,000 to 400,000 human cases of visceral leishmaniasis and 700,000 to 1.2 million human cases of cutaneous leishmaniasis distributed in 98 countries. In the American continent, Brazil is the country with the highest estimated incidences of both visceral and cutaneous leishmaniases [11]. During the past decades, human migrations have resulted in major deforestation and unplanned settlements in Brazil. This has led to the emergence of new transmission profiles of ZCL, driven mostly by human-made environmental changes [12].

In Brazil, seven *Leishmania* species are involved in ZCL transmission [13]. The most widely distributed is *Leishmania (Viannia) braziliensis* (Vianna), recorded in every Brazilian state and causative agent of mucocutaneous leishmaniasis. *Leishmania (Viannia) guyanensis* Floch is also noteworthy, because of its characteristic clinical manifestation with multiple skin lesions. *Leishmania amazonensis* is mainly distributed in the Amazon region. This parasite, when infecting humans, can cause localized cutaneous lesions and eventually develop a more severe clinical form, diffuse cutaneous leishmaniasis (DCL). This clinical form is rare, with chronic development, where the immunodepressed patient shows frequent relapses and insufficient responses to available therapies. Human cases of DCL caused by *L*. *amazonensis* are recorded sporadically in Amazon areas of Brazil, Venezuela, Colombia, Bolivia and Peru. In Brazil, DCL is recorded in North, Northeast, Central West and Southeast regions [14, 15].

*Lutzomyia flaviscutellata* has been incriminated as the vector of *L*. *amazonensis* in Amazonian Brazil [16,17]. It is a sylvatic sand fly that feeds at ground level on a variety of animals including marsupials and birds, but it is most strongly attracted to rodents [18]. In addition to *L*. *flaviscutellata*, there may be as many as five other taxa in the “*L*. *flaviscutellata* complex” [19,20], which share morphological and behavioural characteristics and are implicated in the transmission of *L*. *amazonensis* or closely related *Leishmania* species [9]. *Lutzomyia (Nyssomyia) olmeca olmeca* (Vargas & Nájera) is restricted to Central America; *L*. *(N*.*) olmeca bicolor* Fairchild & Theodor is found in Central America and northern South America; *L*. *(N*.*) olmeca nociva* Young & Arias is restricted to western Amazonian Brazil; *L*. *(N*.*) reducta* Feliciangeli, Ramirez Pérez & Ramirez is restricted to the western Amazon region; and *L*. *(N*.*) inornata* Martins, Falcão & Silva can be considered a synonym of *L*. *flaviscutellata* [20]. In contrast, Galati [21] treated the L. flaviscutellata complex as the genus *Bichromomyia*.

There is evidence that the distribution and population ecology of *L*. *flaviscutellata* are influenced by climate, particularly by seasonal precipitation. In eastern Amazon, for example, this vector was more abundant during the dry season in flooded *Igapó* forests but during the wet season in secondary *Capoeira* forests [22,23]. Its distribution stretches to forest patches and riverine gallery forests in the Brazilian savannah (the *Cerrado* biome) and also in the coastal Atlantic forest [20,21].

Future projections from General Circulation Models (GCMs, models that simulate energy transfer in the atmosphere) indicate that the Amazon region will become progressively drier through strengthening and lengthening of the dry season [24] and the precipitation variability associated with the El Niño-Southern Oscillation (ENSO) will likely intensify [25]. In the last eight years, there have been reports of the first human infected by *L*. *amazonensis* in Rio de Janeiro State, Brazil [26], together with more widespread captures of *L*. *flaviscutellata* to the south of the Amazon region, namely in the Atlantic forest [27,28] and in the *Cerrado* [29–32]. This has prompted the hypothesis that this vector could be expanding its geographical distribution. Ecological niche modelling (ENM) provides a way of exploring the environmental requirements of *L*. *flaviscutellata* and how its distribution might change in response to climate change.

The known occurrences of species can be linked to environmental variation across landscapes in order to estimate ecological niches and geographic distributions. Ecological niche modelling has been widely used in ecology, biogeography and conservation studies, with many published reviews on general applications and specific steps of model development [33–35]. In these models, an algorithm is used to calculate the relationship between species’ occurrence records and environmental variables, in order to create a surface of environmental suitability or probability of species occurrence [36,37]. In climate change studies, after an ENM is critically tested and validated, it can be projected in different time or space, allowing the examination of possible range expansions, contractions or shifts. Discussions of future projections of species’ distributions have to account for variability between different GCMs. Although the use of different GCMs and climate change scenarios can be a great source of variation in ENM, comparative studies demonstrate that most uncertainty is caused by the use of different ENM algorithms [38,39]. Recent comparisons of several niche modelling algorithms concluded that there is not a single approach recommended for every study, and therefore a suite of algorithms should be tested for predictive ability before answering particular questions regarding species niches [40]. In addition to the importance of testing the use of different algorithms, the type and quality of species data strongly influence model outputs. Most ENMs of disease vectors are based on accessible species presence datasets and randomly generated pseudo-absences, instead of carefully selecting absence data in order to significantly improve model performance [41]. The use of species absence data tends to produce better model outputs, which are closely fitted to input data because they can more effectively detect the environmental features discriminating between species presence and absence [42].

Few published studies assessed current and future projections of ecological niches of sand flies using different methods [43–45]. Among South American species, three ZCL vectors in central and southern Brazil–*L*. *(N*.*) whitmani* (Antunes & Coutinho), *L*. *(N*.*) intermedia* (Lutz & Neiva) and *L*. *migonei* (França)—were modelled and the results showed that each should find improving climatic conditions in the future, with *L*. *whitmani* having the largest predicted range expansion [46]. These three sand fly species are involved mostly in the transmission of *Leishmania (Viannia) braziliensis* in long-colonized regions, a parasite species with wider distribution and different epidemiology than *L*. *amazonensis* [10]. Most transmission of *L*. *amazonensis* occurs in the lessdeforested Amazon region. Therefore, it cannot be assumed that *L*. *flaviscutellata* will expand into southeastern Brazil, the most populous region of the country [47], in the same way as predicted for *L*. *whitmani* [46]. The aims of the current study, therefore, were to define the climate niche of *L*. *flaviscutellata* and to use it to explore future projections under climate change scenarios.

## Materials and Methods

### Review of the current distribution

We performed an extensive literature review to compile occurrence records of *L*. *flaviscutellata*. We searched three online databases (PubMed, http://www.ncbi.nlm.nih.gov/pubmed; ISI Web of Knowledge, http://apps.webofknowledge.com and SCOPUS, http://www.scopus.com) during October 2014 using different combinations of the keywords “Psychodidae”, “Lutzomyia” and “flaviscutellata”. We considered as valid records the following species names: *Lutzomyia (Nyssomyia) flaviscutellata*, *Bichromomyia flaviscutellata*, *Phlebotomus flaviscutellatus*, *Flebotomus flaviscutellatus*, *Psychodopygus flaviscutellatus* and *Phlebotomus apicalis*. We also gathered unpublished records from the Health Departments of Brazil and from major Brazilian sand fly collections (Centro de Pesquisas Rene Rachou—FIOCRUZ, Instituto Evandro Chagas—IEC and Faculdade de Saude Publica—USP).

Prior to the descriptions of *L*. *olmeca*, *L*. *olmeca bicolor* and *L*. *olmeca nociva*, all morphologically similar sand flies were identified as *L*. *flaviscutellata*. Articles published up to 1980, therefore, were more carefully reviewed and excluded from the database if cited as having identification errors in later reviews [20,48]. In addition, *L*. *flaviscutellata* only occurs in South America, whereas the range of *L*. *olmeca* stretches northward into Central America and up to Mexico [20,49].

For the ENMs, we also inferred absence records from the literature. The vast majority of the reviewed studies used light traps to capture sand flies. This lowers detectability because *L*. *flaviscutellata* is not as highly attracted to light as many sand fly vectors. The most effective traps for *L*. *flaviscutellata* are rodent-baited “Disney”-like traps [18,50]. Nonetheless, it can be captured in light traps if the local abundance is high enough–usually in the Amazon forest [51,52]–or in long-term monitoring studies in other biomes [28,53]. Therefore, only localities with at least one year of monthly sand fly sampling with no record of *L*. *flaviscutellata*, regardless of the capture method, were considered as absence records.

Species occurrence datasets from secondary data tend to be spatially biased, especially in the Amazon, with records following access points such as roads or rivers [54,55]. Because this could hinder model accuracy, we refined our dataset by removing duplicate records. First, all unique presence and absence points were classified in three categories according to their spatial precision (High: geographical coordinates of capture site given in the reference; Medium: geographical coordinates approximated according to description of capture site; Low: only district or municipality level information, S1 Fig). Then a 20 km buffer was set around each record–if multiple records fell inside the same buffer zone, we retained only the one with the higher spatial precision. The final occurrence dataset used to run the models was composed of 199 presence and 86 absence points (S2 Fig).

### Climate variables

WorldClim (http://www.worldclim.org) provides 19 bioclimatic variables derived from monthly averages of temperature and precipitation [56]. We used a subset of these variables as predictors in the current (average for 1950–2000) and 2050 (average for 2041–2060) projections of *L*. *flaviscutellata* climate suitability. For future conditions we used downscaled and calibrated projections of 17 GCMs (S2 Table) from the fifth assessment report of the International Panel on Climate Change [25], under two different greenhouse gas concentration pathways: “stabilization” (RCP 4.5) and “high increase” (RCP 8.5). These were chosen to represent contrasting scenarios of 21^st^ century climate policies, where radiative forcing of greenhouse gas stabilizes by 2100 (in RCP4.5) or keeps rising after 2100 (in RCP8.5) [25, 57]. All climate data were at the spatial resolution of 10 arc-minutes (ca. 344 km^2^ at the equator). This coarse resolution is compatible with the spatial precision of our *L*. *flaviscutellata* occurrence data. Furthermore, climatic effects on species distributions are better perceived at coarser spatial resolutions [33,58].

To reduce collinearity in the bioclimatic dataset, we selected a subset of less correlated variables. We generated a Pearson correlation matrix (S3 Fig) from the bioclimatic values of *L*. *flaviscutellata*’s records using the package *corrplot* in the software *R* (version 0.73 [59]) and within each pair or group of highly correlated variables (r > 0.6) all but one was removed, with the selection criteria being ecological relevance to the vector. The final set of climate predictors used to run the models consisted of: annual mean temperature (BIO1), mean diurnal range (BIO2), temperature seasonality (BIO4), annual precipitation (BIO12), precipitation seasonality (BIO15) and precipitation of warmest quarter (BIO18).

### Ecological Niche Models

A critical step is the selection of the modelling algorithm, because the use of different methods can lead to different results [40,42,60]. We modelled using two different algorithms for each type of species dataset: presence only (BIOCLIM and DOMAIN), presence/background (MaxEnt and GARP) and presence/absence (GLM and Random Forests). These six algorithms represent different modelling approaches: climate envelope (BIOCLIM), environmental distance (DOMAIN), statistical adjustment (GLM) and machine learning (MaxEnt, GARP and Random Forests).

For the models produced by BIOCLIM and DOMAIN, only the set of 199 presence records of *L*. *flaviscutellata* was used. These presence-only models are developed by constraining the range of environmental predictors to either the minimum and maximum values assigned to all presence records, as in BIOCLIM [61] or by multivariate metrics in environmental space, as in DOMAIN [62].

Presence/background methods estimate potential distributions by comparing the environmental characteristics at sites where the species has been recorded (presence) with those throughout the study region (background). We used MaxEnt, a machine learning algorithm based on maximum entropy [63] and GARP, the genetic algorithm for rule set prediction [64]. For these models, we used the 199 presence records with 10,000 randomly generated background points throughout the study area (South America).

Statistical adjustment and classification algorithms are often used when absence data are available. We used logistic regression, the most common type of Generalized Linear Models (GLM) used in ENM [65] and Random Forests, a machine learning algorithm based on classification of regression trees [66]. GLM and Random Forests models used the full set of 199 presence and 86 absence records.

Most models were developed using the package *dismo* (version 1.0–5 [67]) in the software *R* (version 3.1.1 [68]). GARP models were run in OpenModeller (version 1.1.0 [69]), using its “Best Subsets” new implementation [70]. For every model, we used 10-fold cross validation in order to use the whole set of presence/absence points for both model training and testing. In each model run, 10% of points were randomly selected for model testing. Sixty model runs were performed (10 runs for each one of the six algorithms).

We restricted the model outputs to historically accessible areas to the species via dispersal (M area in the BAM diagram framework [71, 72]). We hypothesized the accessible area of *L*. *flaviscutellata* based on the ecoregions and elevation where it occurs (using data from WWF [73] and WorldClim [56]) and excluding known areas of the vector absence due to major dispersion barriers, such as the Andes [20,49].

The outputs of the algorithms were mapped as continuous values per pixel representing climate suitability. We used standard deviation to compare results from different algorithms and map uncertainty. As the range of values is different for each algorithm, outputs were converted to binary (0 and 1) by applying a threshold, in order to create ensemble maps. We tested two different threshold rules: i) maximization of sensitivity (true positive rate) and specificity (true negative rate), which performs well in evaluations of climate change impacts on species’ ranges [74,75] and ii) zero omission, a more conservative approach which fully maximizes sensitivity while decreasing specificity. We also masked out the predictor values outside the ranges of the input data to avoid strict model extrapolation in the binary predictions of each algorithm, because, for instance, this could include the consideration of high suitability under extreme values unlikely to be biologically realistic [76]. Model significance was evaluated by binomial probabilities calculated over binary outputs, whereas model performance was assessed by True Skill Statistics (TSS) and Cohen’s kappa. Both TSS and kappa range from -1 to +1, where +1 indicates perfect agreement and values of zero or less indicate a performance no better than random [77].

We then produced ensemble maps overlapping the binary projections of the six models with highest TSS and kappa values for each algorithm. Only the areas of agreement of at least four models were retained in the final maps, following the majority ensemble rule [78]. As we opted to include variability of all 17 GCMs, we summed their projection maps for each algorithm. Final consensus maps of current and future predictions were overlapped to visualize expansion and contraction areas of climate suitability in both climate change scenarios.

All binary output maps were projected in the Albers Equal Area Conic coordinate system using the software ArcGIS 10.1. We then calculated the total predicted area of climate suitability for *L*. *flaviscutellata* from the final consensus maps and the changes between current and future predictions. The elevation range of the whole climatically suitable area from the current and future consensus maps was calculated using the Digital Elevation Model available from WorldClim. We sampled elevation values from 10,000 randomly generated points inside the predicted climatically suitable areas, and produced kernel density plots to compare the elevation profiles under current and future scenarios. Wilcoxon rank sum tests were performed to assess statistical differences between each future scenario and the current prediction. Graphics and statistical tests were developed in the software *R*.

## Results

The complete occurrence database of *L*. *flaviscutellata* included 342 presence records. Most of them are from Brazil (277), but other South American countries with records of the species include French Guiana (17), Suriname (15), Colombia (11), Peru (10), Trinidad and Tobago (4), Venezuela (4), Bolivia (2) and Ecuador (2) (S1 Fig, see S1 Table for the gazetteer of occurrence records).

*Lutzomyia flaviscutellata* occurs in areas where the annual mean temperature ranges from 21 to 27.6°C and the annual precipitation varies between 1,139 and 3,843 mm (Table 1). Its current elevation range stretches between 4 and 1,539 m (Table 1).

**Table 1:** Bioclimatic and elevation ranges of occurrence records of *Lutzomyia flaviscutellata*.

	Min.	Median	Mean	Max.
Annual Mean Temperature (°C)	21	26.1	25.6	27.6
Mean Diurnal Range (°C)	6.4	9.8	10.21	15.5
Temperature Seasonality (standard deviation)	2.35	5.53	7.203	28.69
Annual Precipitation (mm)	1139	2109	2089	3843
Precipitation Seasonality (coefficient of variation)	15	58	56.98	94
Precipitation of Warmest Quarter (mm)	19	318	354.2	1034
Elevation	4	134	200.3	1539

**Fig 1:**
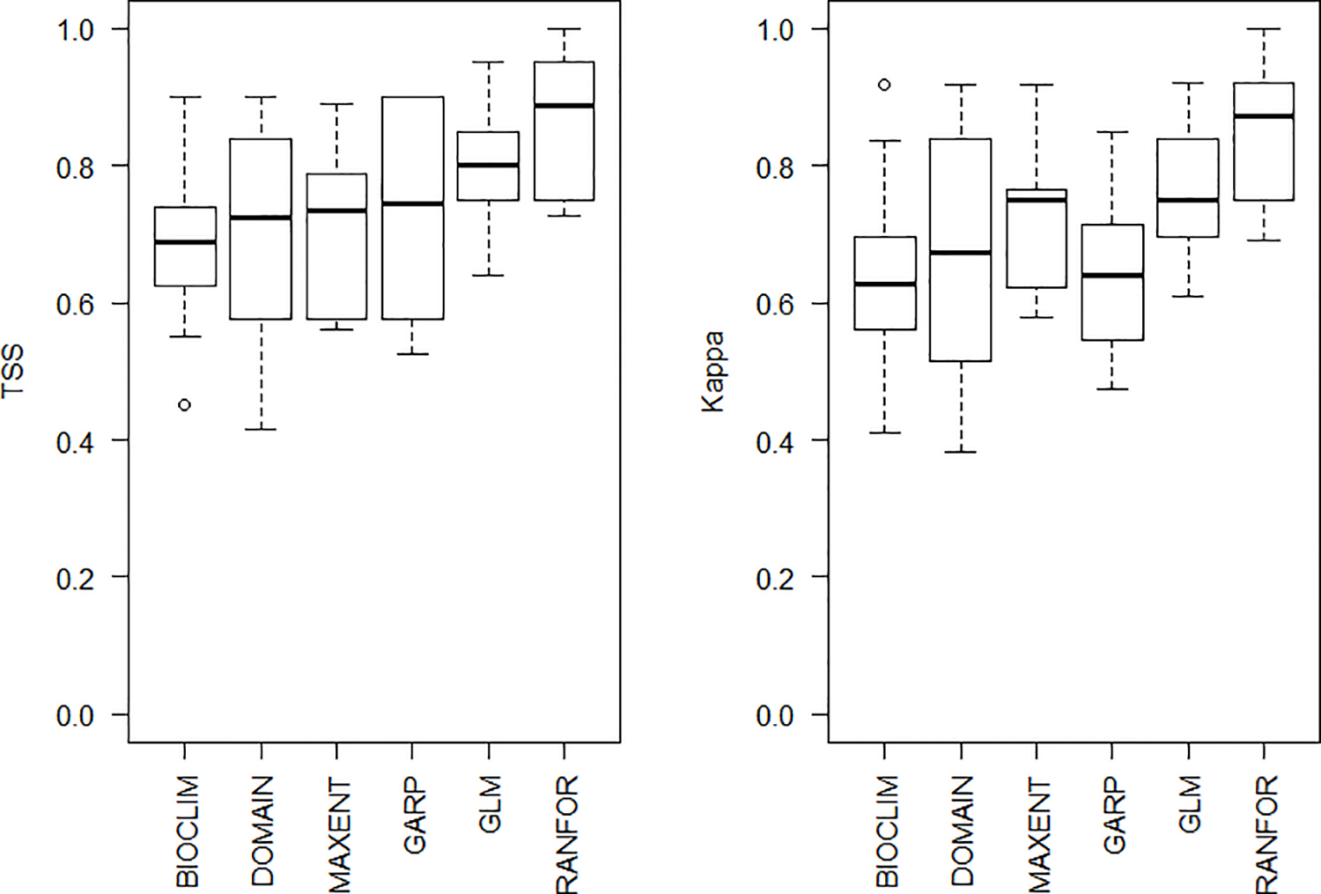
Performance of models produced by the different algorithms according to TSS and Cohen’s kappa.

**Fig 2:**
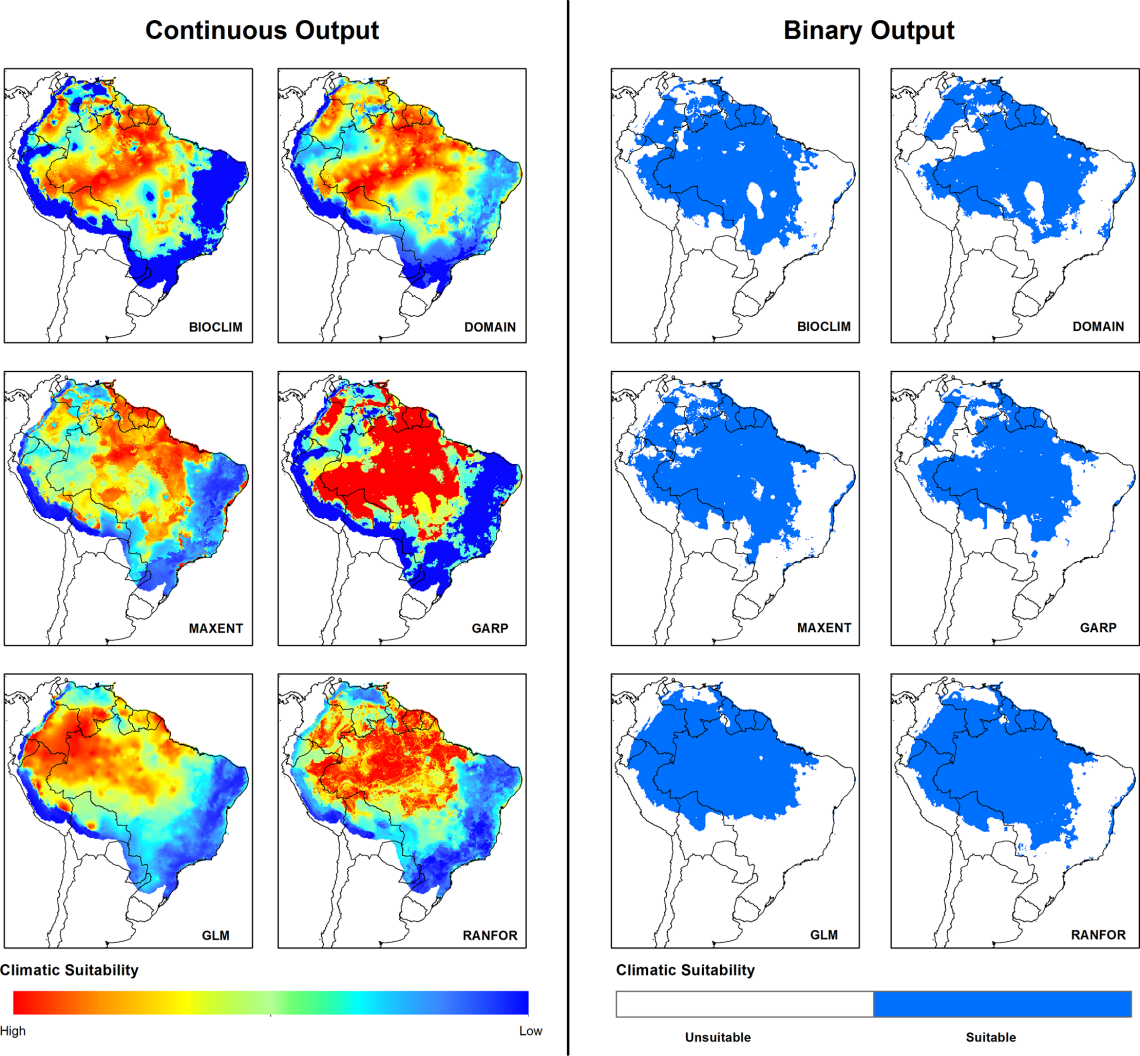
Climate suitability for *Lutzomyia flaviscutellata* in South America under current conditions from six modelling algorithms. Continuous output: stretched values of climate suitability. Binary output: suitable areas after the application of the threshold that maximizes model sensitivity and specificity.

Model performance ranged from fair to excellent (0.4 < TSS > 1; 0.3 < kappa > 1, Fig 1). Outputs with higher values of both TSS and kappa were selected to produce the ensemble models; all of them were significantly better than random predictions (binary probabilities, p< 0.001). For predictions under current climatic conditions, the different algorithms showed a common general pattern with some regional variation (Fig 2). Testing different threshold rule methods showed differences in binary outputs, more evidently in DOMAIN and GLM, while in Random Forests the difference could barely be noticed (S4 Fig). Masking out the predictor values outside the ranges of the input variables showed that models produced by all algorithms had little to no areas of model extrapolation (S5 Fig).

**Fig 3:**
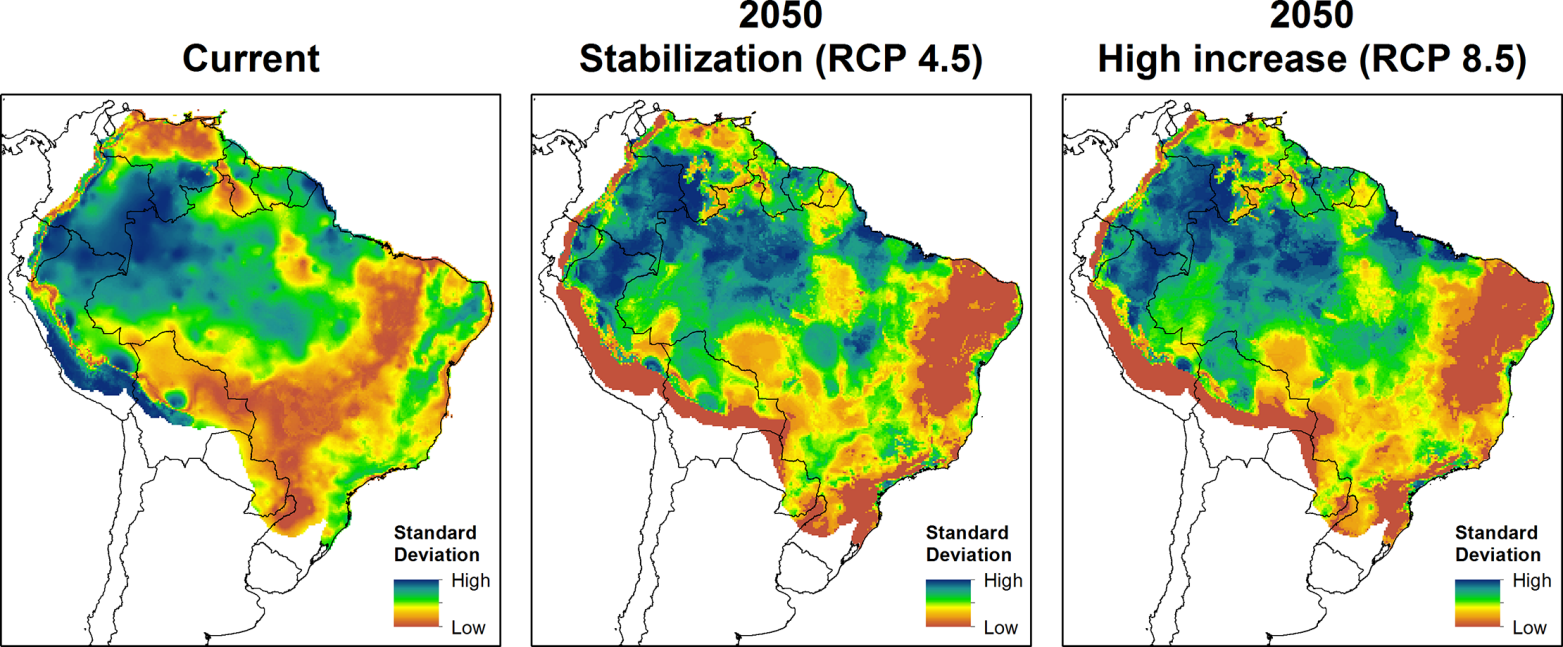
Uncertainty mapping for ecological niche models of *L*. *flaviscutellata*.

**Fig 4:**
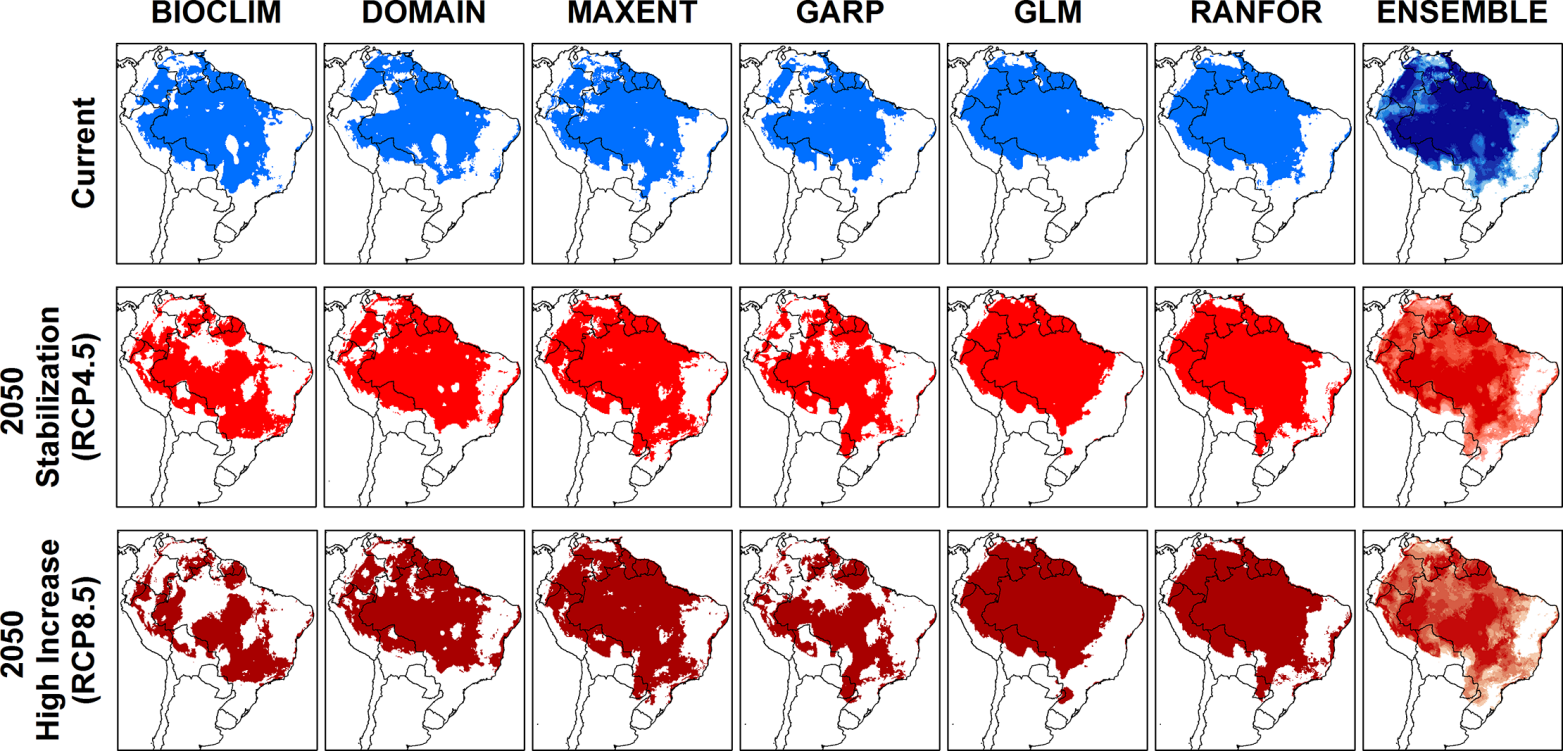
Current and future climate suitability for *Lutzomyia flaviscutellata* from six modelling algorithms and ensemble maps.

Under current climate conditions, all six algorithms predict most of the Amazon as climatically suitable (Fig 2). Mapping the uncertainty between models (Fig 3) showed that the northwestern region of the continent (most of Colombia, southern Venezuela, northern Peru and northwestern Brazil) were areas of disagreement between models. This becomes clearer in the ensemble outputs, where lighter shades of blue and red indicate fewer consensus between models (Fig 4).

Projections under climate change conditions showed quite different results for each of the 17 GCMs, although there was more variation between different ENM algorithms than between different GCMs (S6, S7, S8 and S9 Figs). When combined, however, most algorithms predicted an expansion of the total area of climate suitability of *L*. *flaviscutellata* (Fig 4). All models agree that the species should find improving climate conditions towards the southern limits of its distribution, especially in the “high increase” scenario (RCP 8.5).

**Fig 5:**
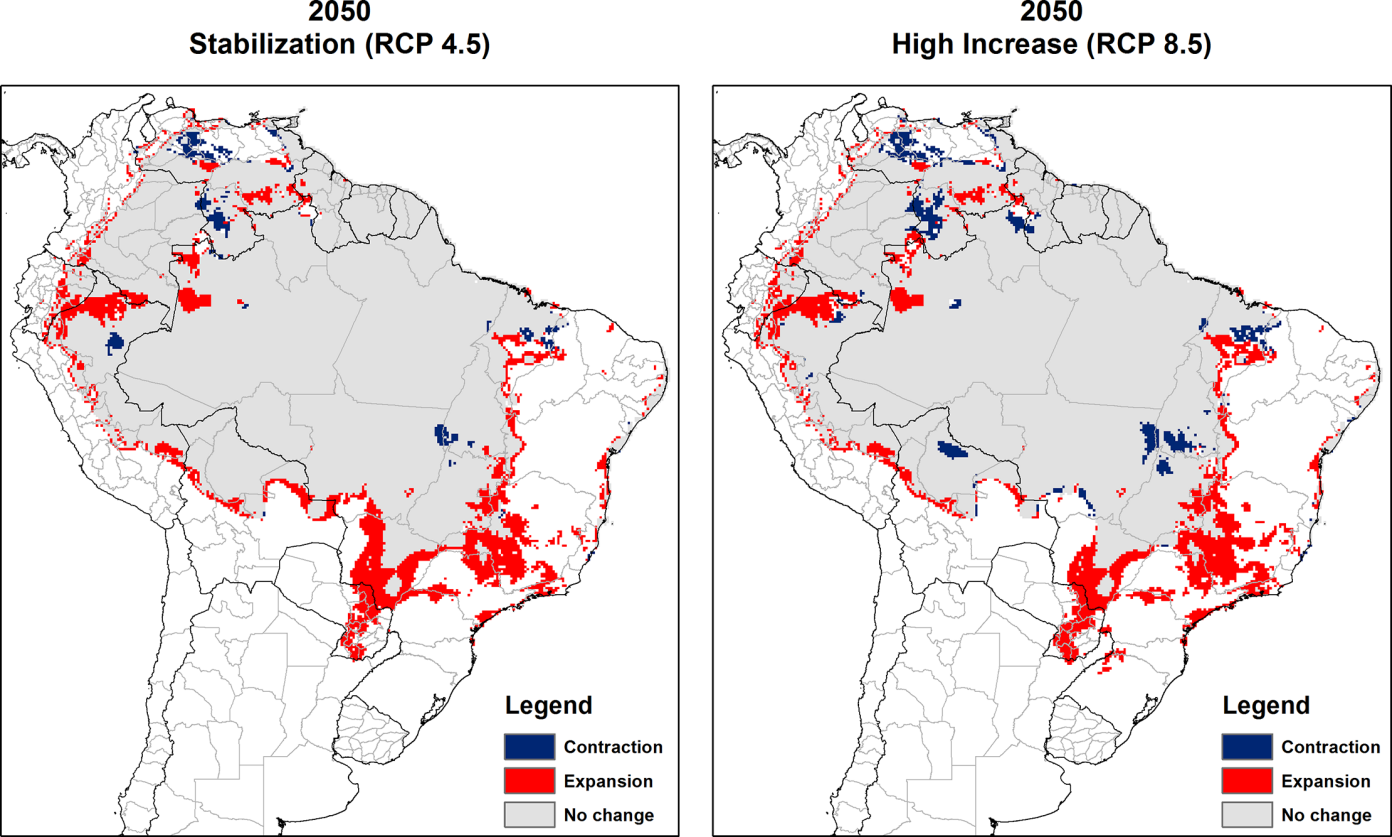
Consensus maps of predicted future climate suitability of *Lutzomyia flaviscutellata*. Left: Stabilization climate scenario (RCP4.5); right: High increase climate scenario (RCP8.5). Future expansion areas (red), future contraction (blue) and no change between current and future climate suitability (grey).

The final consensus maps for both scenarios predict the expansion of the southern limits of the climatically suitable area for *L*. *flaviscutellata* (Fig 5), including most notably the Brazilian states of Minas Gerais, Mato Grosso do Sul, São Paulo, Amazonas and Maranhão. Other major expansion areas include eastern Paraguay and Loreto Department in Peru. Some minor contraction is also projected in specific areas of central Brazil, Venezuela and Peru.

**Fig 6:**
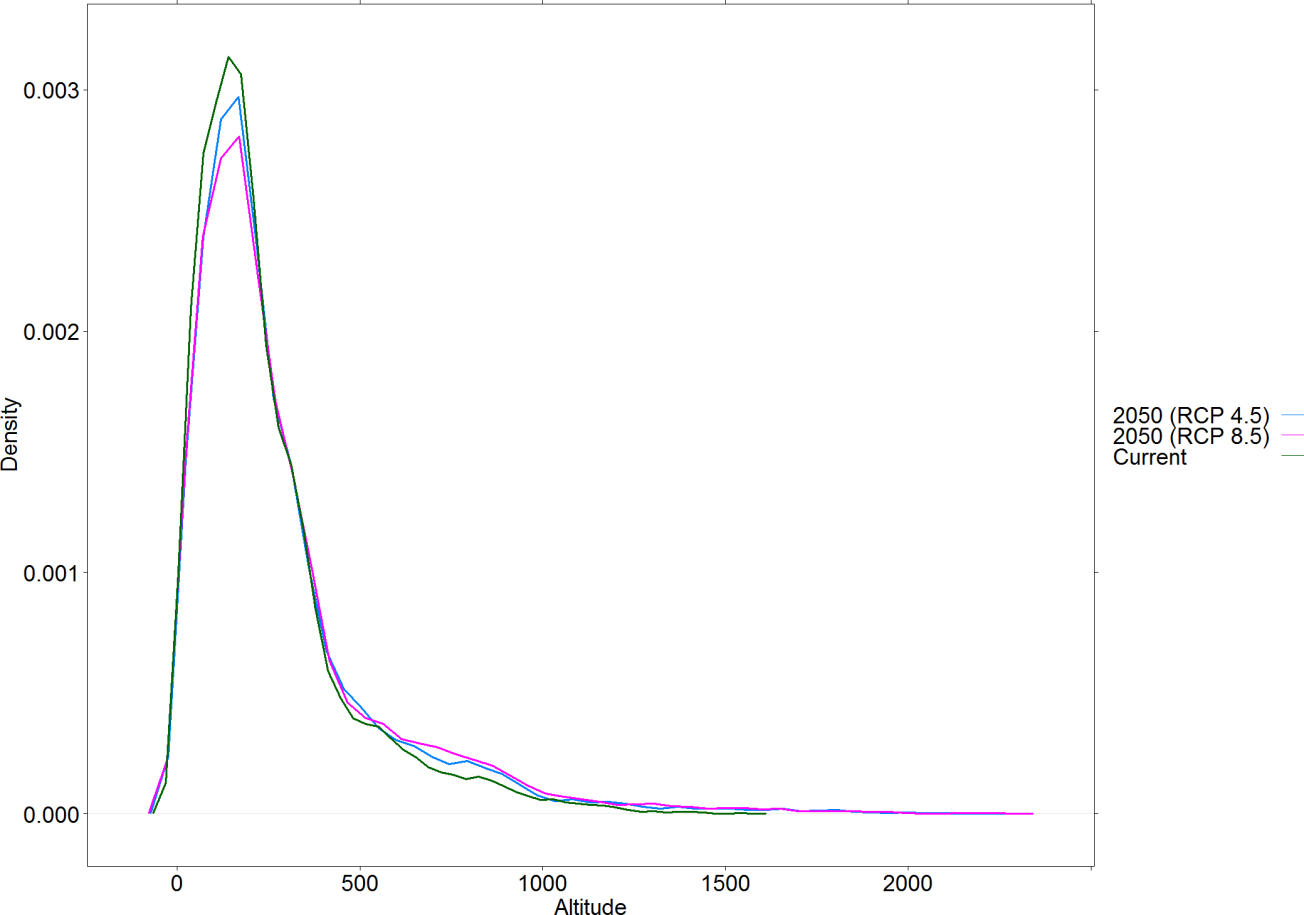
Elevation profiles of current and future projections of climate suitability of *Lutzomyia flaviscutellata*.

The final predicted climate suitability area for *L*. *flaviscutellata* increased by 12.8% in the “stabilization” scenario and by 10.7% in the “high increase” scenario when compared with current predictions (Table 2). There were significant changes in the predicted elevation profile of the species (Fig 6), with the maximum elevation value increasing from 1,545 m to 2,213 m in the “stabilization” scenario and to 2,265 m in the “high increase” scenario (Table 2).

**Table 2:** Predicted area of climate suitability and elevation ranges of *Lutzomyia flaviscutellata* calculated from binary predictions of final consensus maps.

	Area (km²)		Elevation (m)				
	Total	Difference	Min.	Median	Mean	Max.	Difference*
Current	8,126,549	-	0	185	242.9	1,545	-
2050 (“stabilization” scenario)	9,165,933	+12.8%	0	197	277.6	2,213	W = 47,022,356 *p*<0.001
2050 (“high increase” scenario)	8,991,938	+10.7%	0	202	287.7	2,265	W = 46,296,853 *p*<0.001

*Statistical significance in elevation difference defined from Wilcoxon rank sum tests between each future scenario and the current prediction.

## Discussion

### Model predictions and uncertainty

The performance testing of different algorithms followed by selection of the best ones in a consensus is becoming the norm [40,78,79], because the use of different modelling methods may lead to contrasting results [38,39,80]. Presence only models are more mathematically simple and therefore our models produced by BIOCLIM and DOMAIN showed the widest variation in predictive performance. Presence/background methods MaxEnt and GARP had good and similar performances, although MaxEnt had a shorter TSS variability between its outputs. Therefore, MaxEnt would be the algorithm with the best results if we did not have any information on absence records of *L*. *flaviscutellata*. This agrees with a comparative study of the predictive power of several modelling algorithms, where MaxEnt was one of the best among methods that do not use absence data [80]. MaxEnt has been a popular method in recent years [43–45,81–83], possibly because of its easy interface and good performance. Random Forests models had the best performance, which agrees with a modelling exercise of *Culicoides imicola* Kieffer, vector of Bluetongue virus in Spain, where it outperformed GLM and discriminant analysis [41]. Our results showed that the inclusion of species absence data greatly improves model performance, which is in accordance with recent ENM studies [41, 42]. Absence data, however, can also be a source of bias in models if not treated correctly. An absence record may be false if the studied species has low detectability, which could happen for reasons such as low abundance, marked seasonality or lack of an optimal capture method. Even if a species is really absent from a surveyed region, this might be explained by reasons other than lack of environmental suitability, such as dispersal limitations, historical factors or biotic interactions [84]. This is why we used a very conservative criterion for selecting absence records of *L*. *flaviscutellata*. Several locations were surveyed for sand flies without the detection of *L*. *flaviscutellata*, but they were not used for modelling because, being mostly sporadic captures, they did not provide the sampling effort needed to detect the fly’s presence.

Variation from different GCMs were expected because there is an inherent uncertainty in forecasting anthropogenic climate change [38,85]. However, comparative studies demonstrate that the use of different ENM algorithms, rather than different GCMs, causes most model uncertainty [38,39]. This was shown in individual predictions by ENM algorithms and GCMs (S6, S7, S8 and S9 Figs), where outputs from the same algorithms are more similar to each other than outputs from different ones. The 17 GCMs used here are the most up-to-date, from the phase 5 of the Coupled Model Intercomparison Project [25]. For earlier sets of GCMs, comparative studies demonstrated that HadCM3 had very good representation of the South American climate [86]. However, the latest GCMs have only recently become available and their regional variation is yet to be fully explored. Future attempts to improve projections of species distributions based on climate models would benefit from assessments of regional performance of GCMs.

Future projections from our models indicate that the climatically suitable area for *L*. *flaviscutellata* should expand mainly southeastward and southward towards higher latitudes. Other ZCL vectors from South America showed similar results, including *L*. *whitmani* [46], as well as other sand flies from Central and North America [43,45]. Both climate change scenarios also indicate that some parts of the Amazon (mainly west and central) should become less climatically suitable for *L*. *flaviscutellata*, which might be associated with the region’s predicted reduced precipitation in future decades [24]. ENMs combined with climate change predictions also demonstrated some loss of suitable environments in the Amazon for *L*. *whitmani* and *L*. *intermedia* [46]. However, these two species occur mainly in the Brazilian savannah and the Atlantic forest, and they are not as widespread in the Amazon as *L*. *flaviscutellata*.

In the “high increase” scenario, we expected the total area of climate suitability to be higher than in the “stabilization” scenario, but we found the opposite result (Table 2). This might be an indication that moderate changes in precipitation and temperature may be beneficial to *L*. *flaviscutellata*, whereas strong changes would be harmful. This type of response to different scenarios of climate change was observed for other ecological systems, such as the effect of tree species composition on forest net primary production [87]. The increase in the upper elevation bound predicted by both climate change scenarios (Table 2) suggests that the species could shift its elevation range upwards. This was empirically demonstrated for *Phlebotomus ariasi* Tonnoir from the Madrid region, Spain [88,89]. Future elevation shifts were also predicted for *Lutzomyia (Lutzomyia) longipalpis* (Lutz & Neiva) and *L*. *evansi* (Nuñez-Tovar), vectors of visceral leishmaniasis in Colombia [90].

### Public health priorities and future research

Knowledge about vector distributions is essential for understanding leishmaniasis eco-epidemiology and for the success of control and surveillance activities. Our results include the updated geographical distribution of *L*. *flaviscutellata*, main incriminated vector of *L*. *amazonensis* in South America. This new list of occurrence locations (S1 Table) can support Health Departments for the planning of surveillance activities. The updated distribution, however, does not substantially change the previously known country range of the species according to Young & Duncan [20]. A few recent records of *L*. *flaviscutellata* are outside the boundaries of the previously known distribution, such as in Mato Grosso do Sul, Brazil [91,92], in Cusco, Peru [93] and in Orellana, Ecuador [94]. The species has been recorded in almost every South American country north of the Equator except Guyana, although it is likely to occur there based on all model outputs (Fig 2). In Guyana and elsewhere in South America, the detection of *L*. *amazonensis* in human cases should ideally be followed up by vector surveys using rodent-baited traps, such as modified Disney traps [50].

There are recent records of *L*. *flaviscutellata* in southeastern Brazil [27,28,53,95]. It is nearly impossible, however, to test the hypothesis of a recent expansion of *L*. *flaviscutellata* distribution associated with climate change, because of insufficient earlier sampling to demonstrate the species’ prior absence in some regions. Both the coastal Atlantic forest and savannah regions in Southeast and Central Brazil are already climatically suitable for *L*. *flaviscutellata* according to our models for current conditions. This result could be interpreted as a refutation of the hypothesis of a recent distribution expansion associated with climate change. However, the recent records of the species in the Atlantic forest and Brazilian savannah biomes were incorporated into the model, assuming that they represent part of the historical distribution of the species. Because these records were included in model building, the classification of the region where they occur as climatically suitable under current conditions was expected, and could be a methodological artefact. However, the emergence of transmission of *L*. *amazonensis* could be a proxy for the vector’s expansion, such as the recent records in Paraty, southeast Brazil [26] and in Serra da Bodoquena region, central-west Brazil [91,96]. At the same time, there is growing evidence that *L*. *flaviscutellata* can be found near human dwellings in rural areas. The species has been recently captured in peridomestic areas outside the Amazon forest [28,32,97] and even in peri-urban areas [29,30,95,98]. The ability of *L*. *flaviscutellata* to survive deforestation and rapidly colonize secondary habitats has been demonstrated near the Brazilian city of Belém [22] as well as in plantations of exotic trees in the eastern Amazon [23]. This suggests that, even if *L*. *flaviscutellata* does not fully expand its distribution to the predicted future areas of climate suitability, it may colonize areas of recent deforestation at a local scale and thus increase the local risk of human exposure to *L*. *amazonensis*. The inclusion of land cover variables in our models would likely have reduced the biotic uncertainty of our predictions at a local scale. Nevertheless, the decision to use only climate variables was justified because of the continental scale and relatively low resolution of the current study.

Our models do not include information on the occurrence of the parasite *L*. *amazonensis*. Some correlations between ENSO and increases in leishmaniasis have been demonstrated [99–102]. Because future forecasts suggest an intensification of ENSO-related precipitation variability [25], ZCL transmission in the Amazon might increase due to climate change, regardless of the likely changes in the distribution of its vectors. Surveillance for infections of *L*. *amazonensis* is difficult, because identification of the parasite to species is not routine. Inclusion of data on parasite occurrence would improve our ability to predict risk areas for human infection, for which information on the distribution of competent reservoir hosts would also be required, as well as a mechanistic, process-based modelling of the transmission dynamics [8,103].

The current results raise awareness of the predicted expansion of *L*. *flaviscutellata* near the borders of the Amazon–in Bolivia, Peru, Colombia and Venezuela–as well as many parts of Minas Gerais and São Paulo states, in Southeast Brazil (Fig 5). The resident population of these two states is approximately 60.8 million people, more than twice the 25.4 million people living in all the Amazonian states of Brazil [47], where most recorded transmission of *L*. *amazonensis* currently occurs. In fact, there are two relatively recent records of *L*. *amazonensis* infections in domestic dogs in both states, Minas Gerais [104] and São Paulo [105]. The predicted expansion of the area of climate suitable for *L*. *flaviscutellata* in Maranhão state has the potential to significantly increase the prevalence of DCL caused by *L*. *amazonensis*, because this form of the disease is associated with the state [14]. This parasite species has also been recorded sporadically in Paraguay, but not *L*. *flaviscutellata* [9,106]. The elevation range of *L*. *flaviscutellata* could increase as predicted, although vector abundances might well remain too low to permit establishment of new *L*. *amazonensis* transmission cycles. At high elevation, such as the Andes region, sand fly diversity is lower and leishmaniasis transmission is sustained by a few dominant vectors [107,108]. If transmission cycles of *L*. *amazonensis* driven by the dispersion of the vector *L*. *flaviscutellata* establish in these regions, more people will be at risk of acquiring the disease.

Our large-scale study serves as a base for future studies exploring factors that constrain the distribution of *L*. *flaviscutellata* at finer scales, which is a necessary contribution to Public Health research and interventions aimed at reducing the disease burden. We conclude that this vector might well find improving climate conditions for its expansion in the approaching decades, although these new areas will only become endemic for the transmission of *L*. *amazonensis*, if reservoir host populations are present and transmission dynamics are sufficient. In Southeast Brazil, at least, this is already happening [26,104,105, 109].

## Supporting Information

S1 Fig Presence and absence locations of *Lutzomyia flaviscutellata* classified according to spatial precision. High: geographical coordinates of capture site given in the published article; Medium: geographical coordinates approximated according to description of capture site; Low: only district or municipality level information.
(TIF)

S2 Fig Set of presence and absence records of *Lutzomyia flaviscutellata* used to run the models. (TIF)

S3 Fig Pearson correlation matrix of the 19 bioclimatic variables. bio1: annual mean temperature; bio2: mean diurnal range; bio3: isothermality; bio4: temperature seasonality; bio5: max temperature of warmest month; bio6: min temperature of coldest month; bio7: temperature annual range; bio8: mean temperature of wettest quarter; bio9: mean temperature of driest quarter; bio10: mean temperature of warmest quarter; bio11: mean temperature of coldest quarter; bio12: annual precipitation; bio13: precipitation of wettest month; bio14: precipitation of driest month; bio15: precipitation seasonality; bio16: precipitation of wettest quarter; bio17: precipitation of driest quarter; bio18: precipitation of warmest quarter; bio19: precipitation of coldest quarter.
(TIFF)

S4 Fig Binary outputs of current predictions of climate suitability for *Lutzomyia flaviscutellata* using two different threshold rules. (TIF)

S5 Fig Masked outputs of current predictions of climate suitability for *Lutzomyia flaviscutellata*. (TIF)

S6 Fig Current and 2050 (RCP 4.5) projections of climate suitability for *Lutzomyia flaviscutellata* from six modelling algorithms and 17 General Circulation Models, part 1 of 2. Each map shows binary model outputs. Future projections include the percentage of area lost or gain in comparison with current predictions. For names of each General Circulation Model, see S2 Table.
(TIF)

S7 Fig Current and 2050 (RCP 4.5) projections of climate suitability for *Lutzomyia flaviscutellata* from six modelling algorithms and 17 General Circulation Models, part 2 of 2. Each map shows binary model outputs. Future projections include the percentage of area lost or gain in comparison with current predictions. For names of each General Circulation Model, see S2 Table.
(TIF)

S8 Fig Current and 2050 (RCP 8.5) projections of climate suitability for *Lutzomyia flaviscutellata* from six modelling algorithms and 17 General Circulation Models, part 1 of 2. Each map shows binary model outputs. Future projections include the percentage of area lost or gain in comparison with current predictions. For names of each General Circulation Model, see S2 Table.
(TIF)

S9 Fig Current and 2050 (RCP 8.5) projections of climate suitability for *Lutzomyia flaviscutellata* from six modelling algorithms and 17 General Circulation Models, part 2 of 2. Each map shows binary model outputs. Future projections include the percentage of area lost or gain in comparison with current predictions. For names of each General Circulation Model, see S2 Table.
(TIF)

S1 Table *Lutzomyia flaviscutellata* occurrence database. (XLSX)

S2 Table General Circulation Models used in the models. (XLSX)
