# Supplementary figures and images for: Ecological Niche Modelling Predicts Southward Expansion of Lutzomyia (Nyssomyia) flaviscutellata (Diptera: Psychodidae: Phlebotominae), Vector of Leishmania (Leishmania) amazonensis in South America, under Climate Change
Source: PLoS One. 2015 Nov 30;10(11):e0143282. doi: 10.1371/journal.pone.0143282 (PMC4664266; doi:10.1371/journal.pone.0143282)

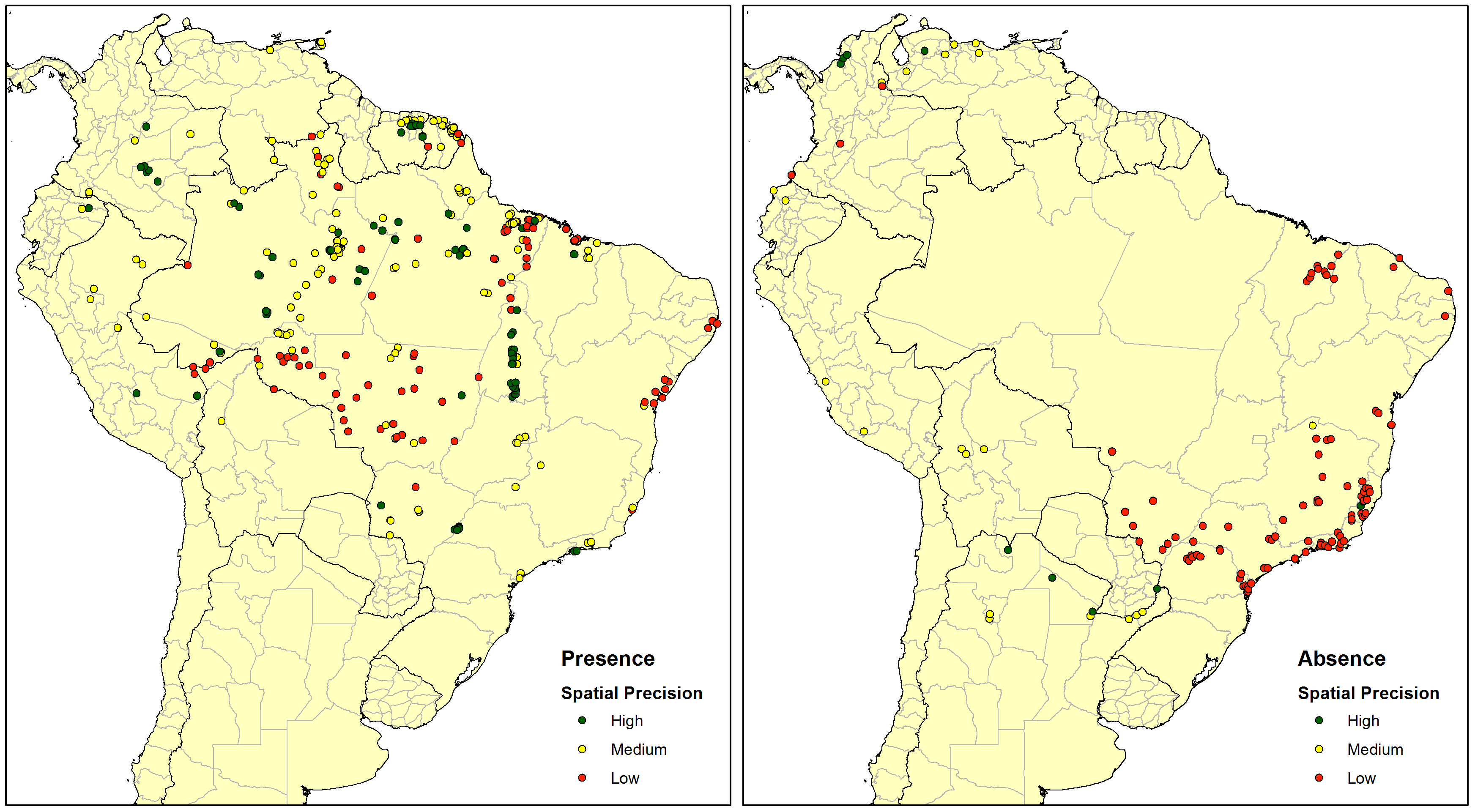

Supplement: S1 Fig — High: geographical coordinates of capture site given in the published article; Medium: geographical coordinates approximated according to description of capture site; Low: only district or municipality level information. (TIF) [file pone.0143282.s001.tif]

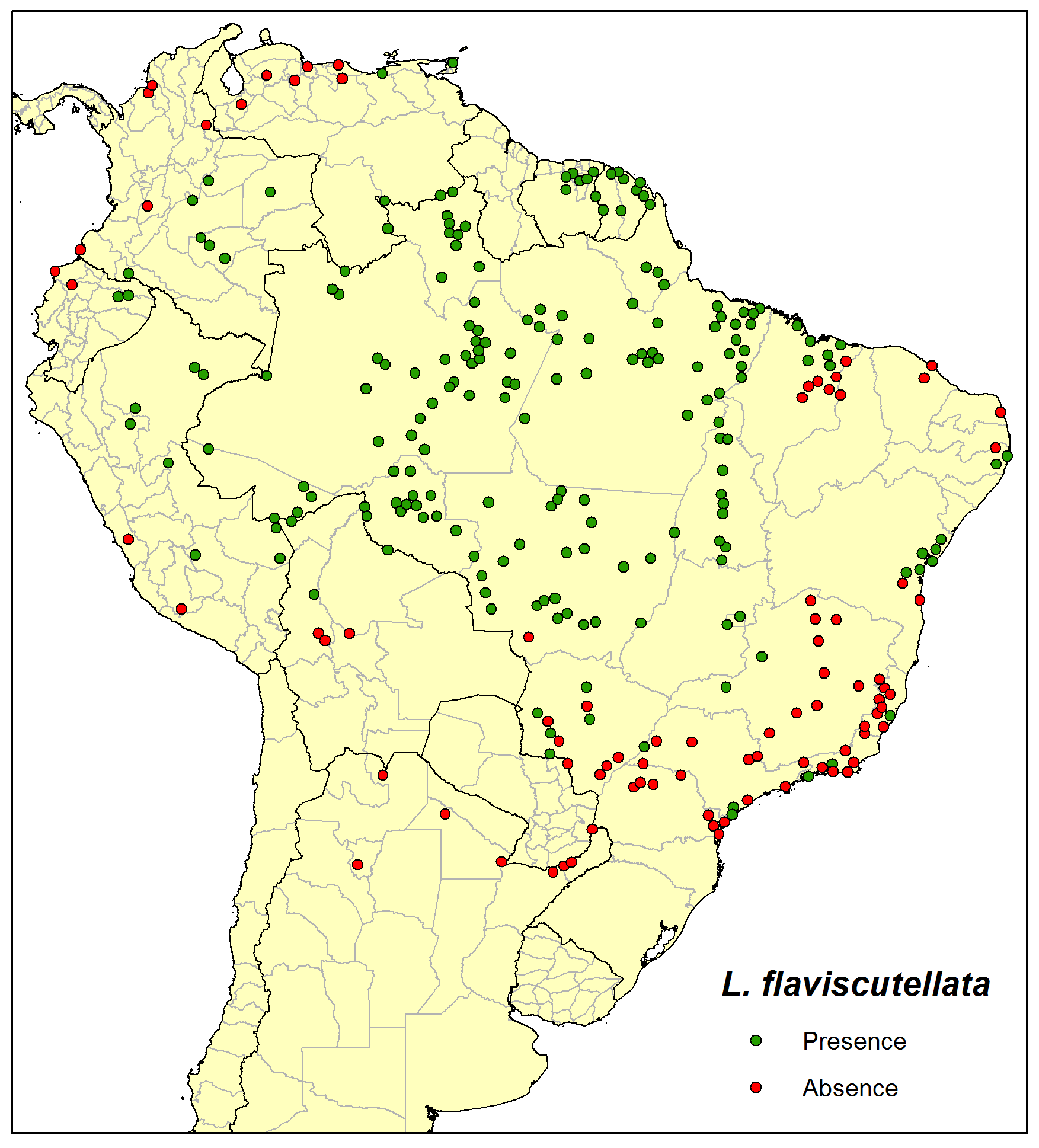

Supplement: S2 Fig — (TIF) [file pone.0143282.s002.tif]

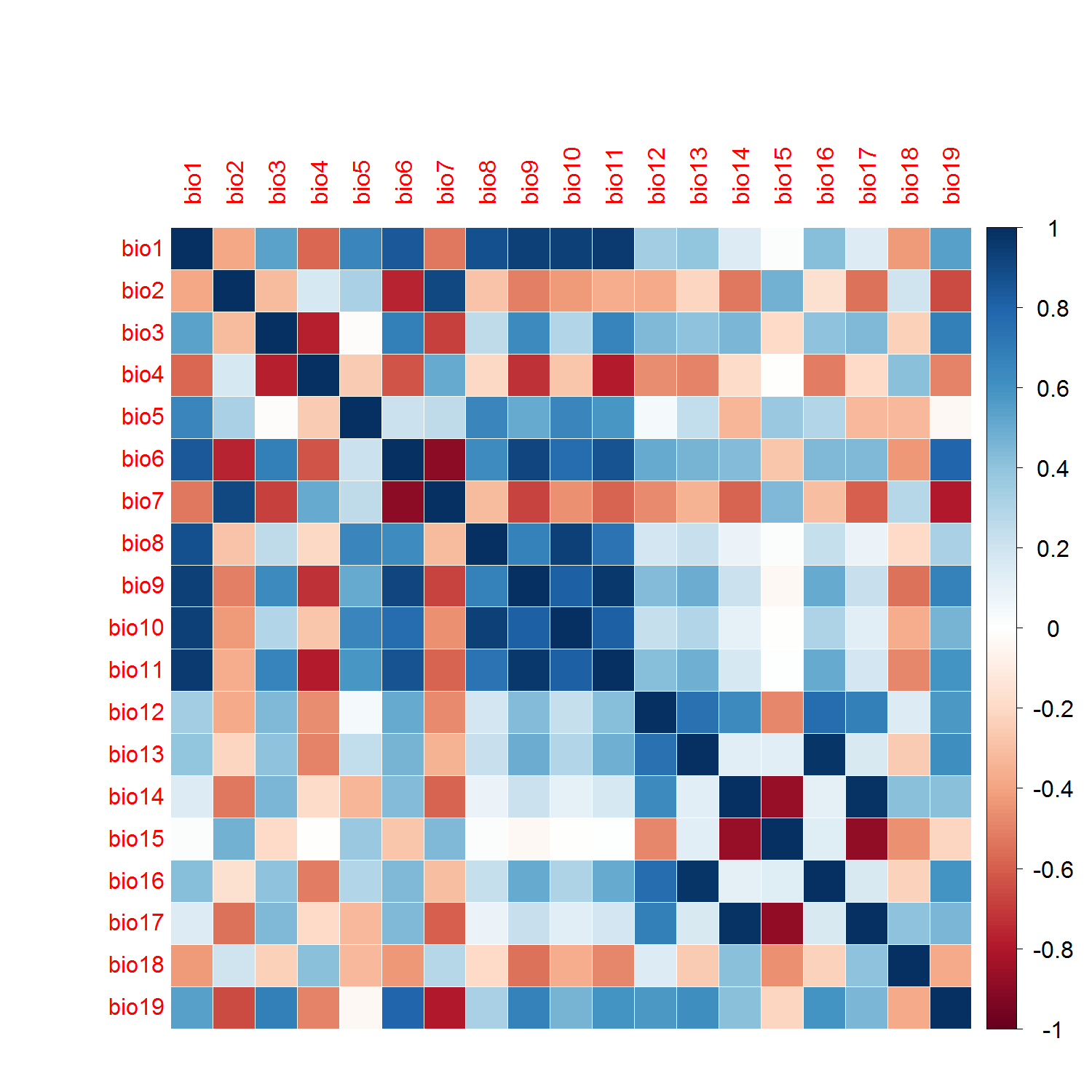

Supplement: S3 Fig — bio1: annual mean temperature; bio2: mean diurnal range; bio3: isothermality; bio4: temperature seasonality; bio5: max temperature of warmest month; bio6: min temperature of coldest month; bio7: temperature annual range; bio8: mean temperature of wettest quarter; bio9: mean temperature of driest quarter; bio10: mean temperature of warmest quarter; bio11: mean temperature of coldest quarter; bio12: annual precipitation; bio13: precipitation of wettest month; bio14: precipitation of driest month; bio15: precipitation seasonality; bio16: precipitation of wettest quarter; bio17: precipitation of driest quarter; bio18: precipitation of warmest quarter; bio19: precipitation of coldest quarter. (TIFF) [file pone.0143282.s003.tiff]

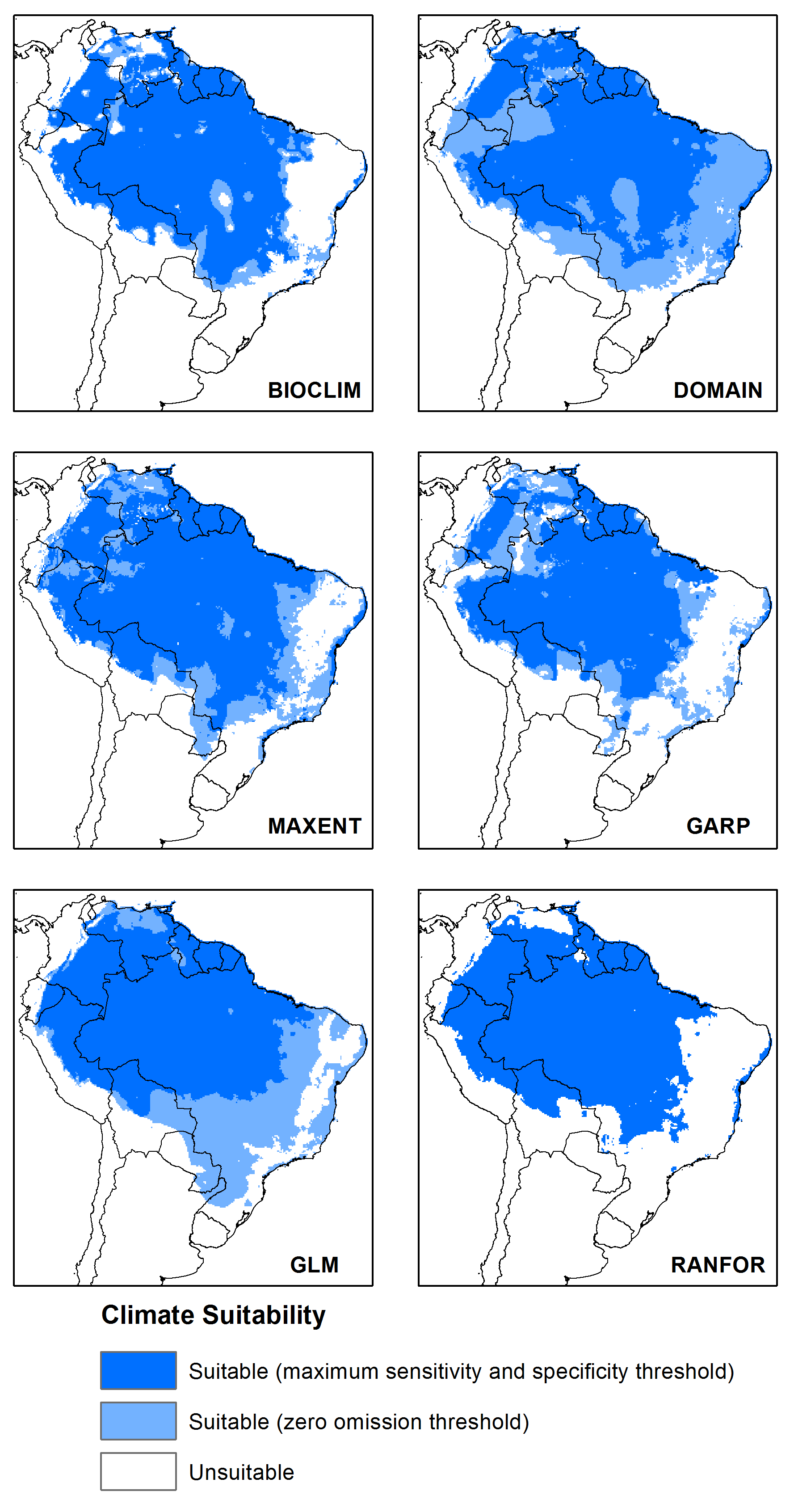

Supplement: S4 Fig — (TIF) [file pone.0143282.s004.tif]

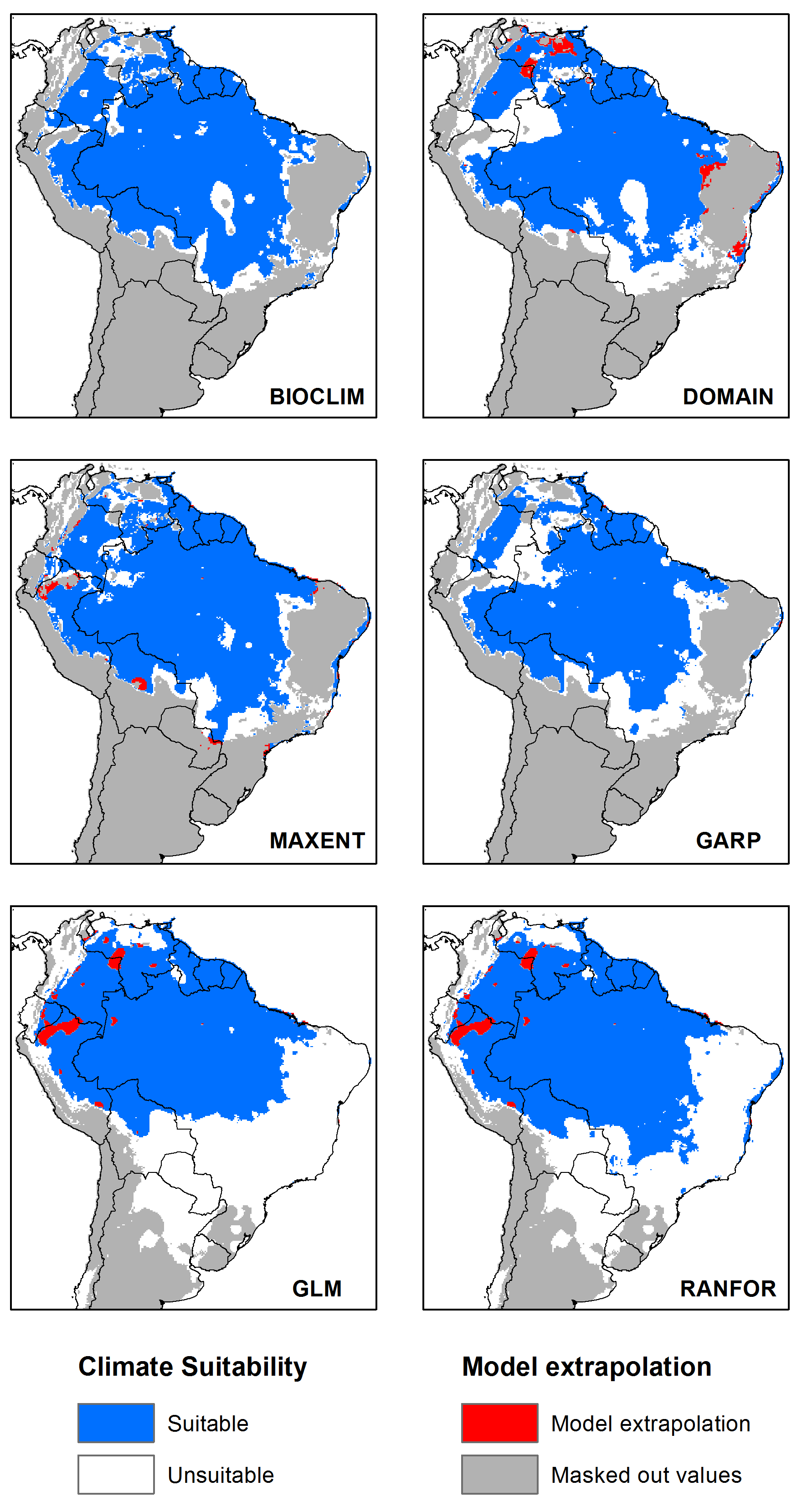

Supplement: S5 Fig — (TIF) [file pone.0143282.s005.tif]

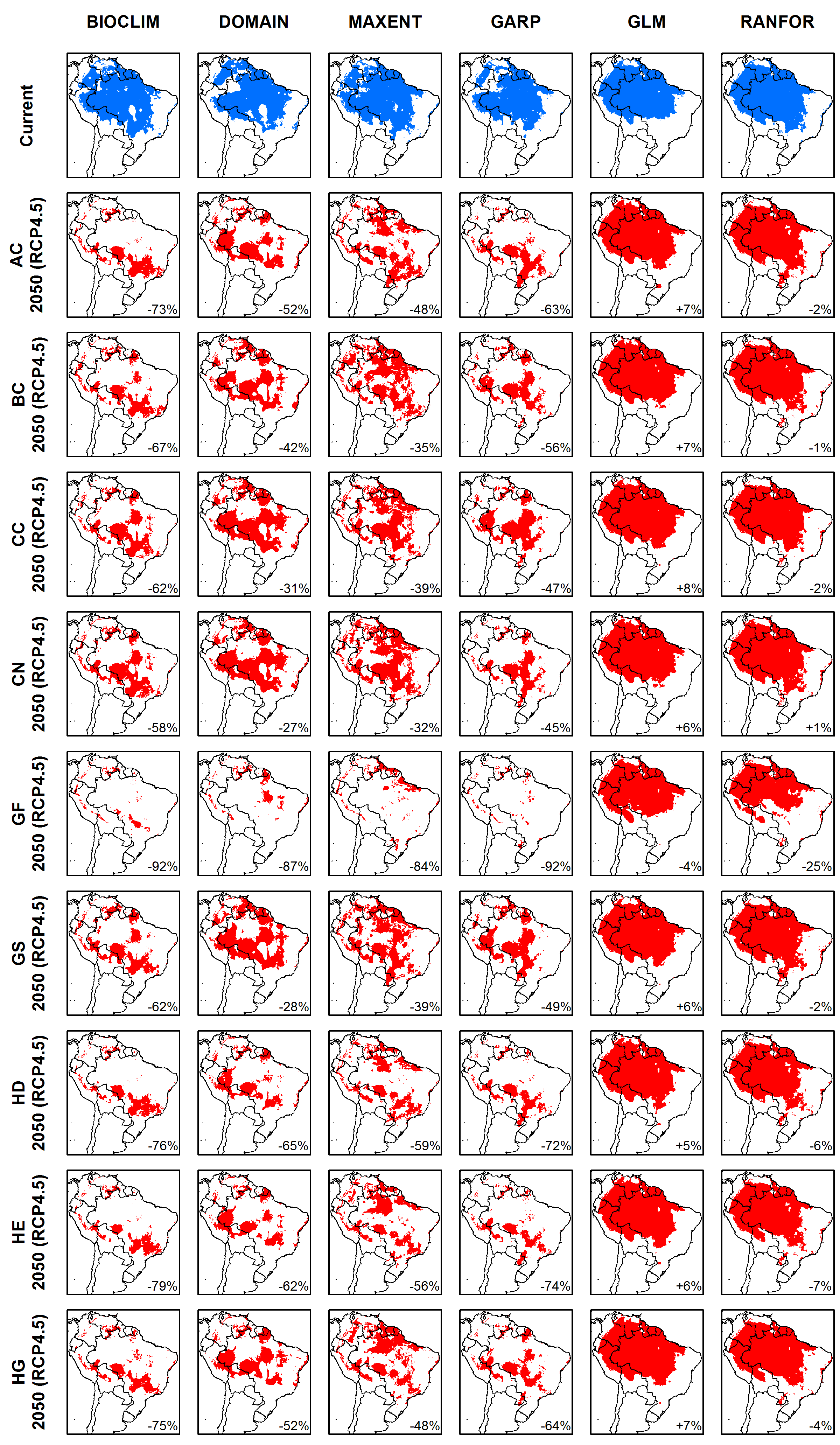

Supplement: S6 Fig — Each map shows binary model outputs. Future projections include the percentage of area lost or gain in comparison with current predictions. For names of each General Circulation Model, see S2 Table. (TIF) [file pone.0143282.s006.tif]

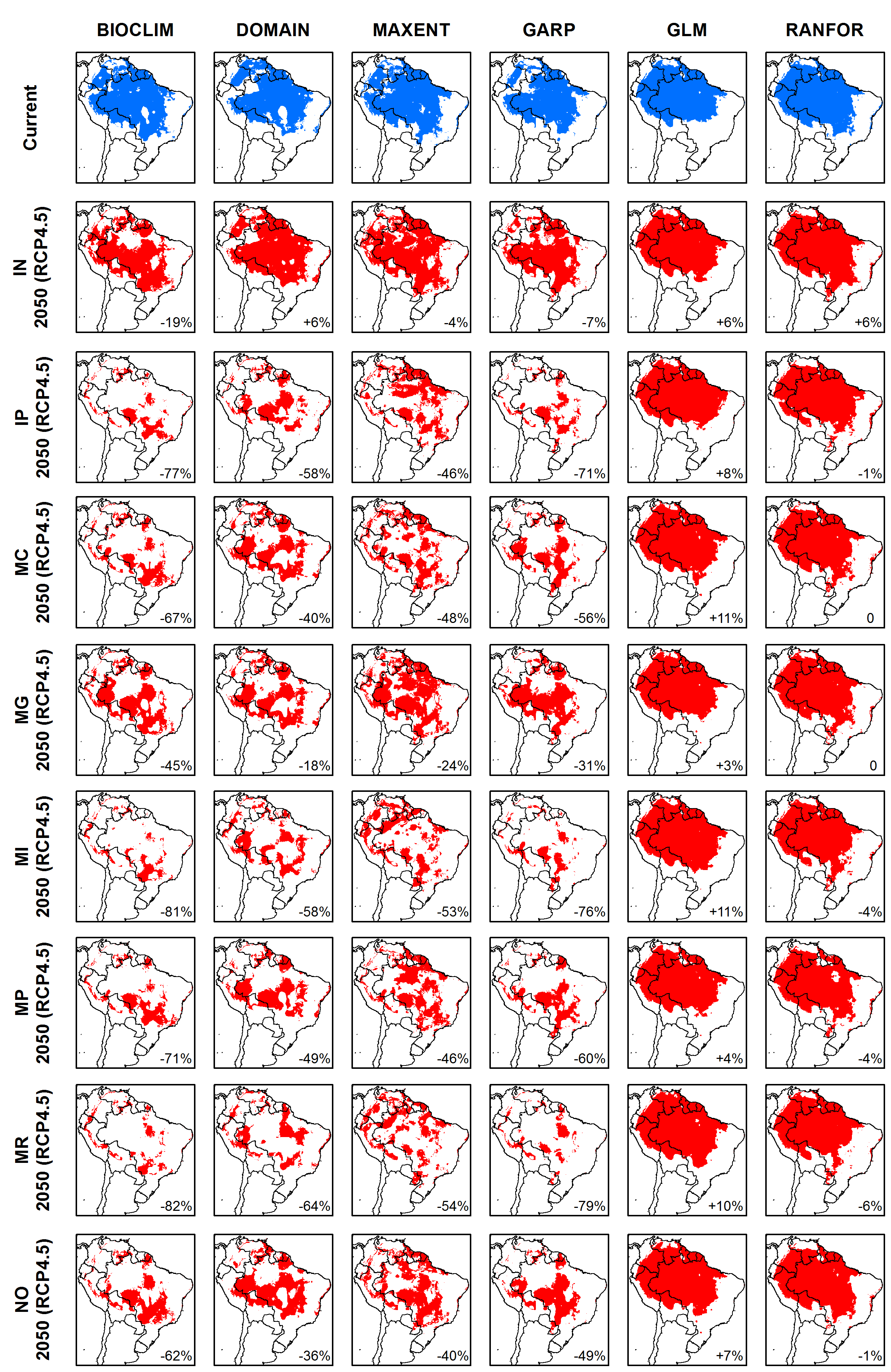

Supplement: S7 Fig — Each map shows binary model outputs. Future projections include the percentage of area lost or gain in comparison with current predictions. For names of each General Circulation Model, see S2 Table. (TIF) [file pone.0143282.s007.tif]

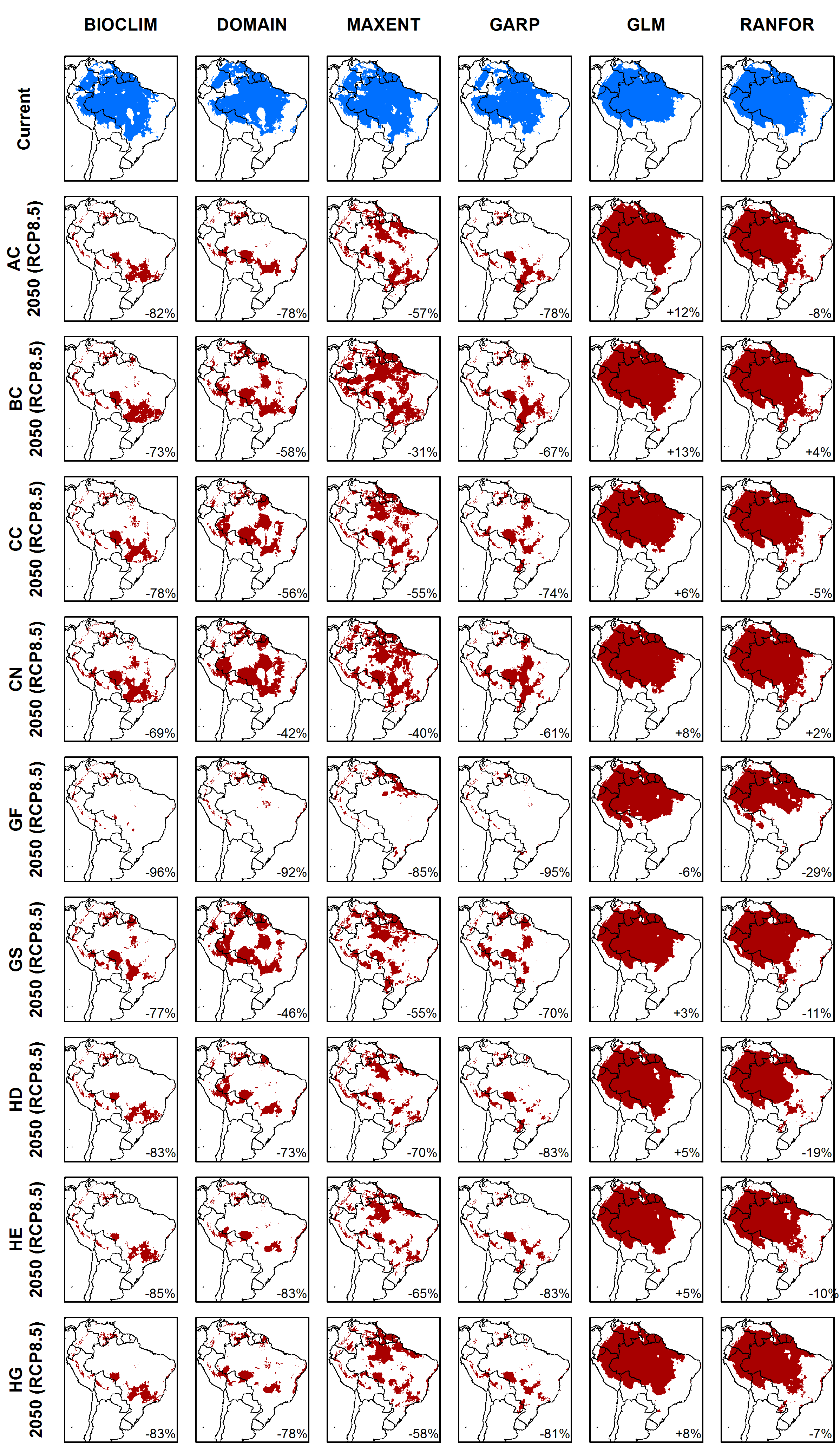

Supplement: S8 Fig — Each map shows binary model outputs. Future projections include the percentage of area lost or gain in comparison with current predictions. For names of each General Circulation Model, see S2 Table. (TIF) [file pone.0143282.s008.tif]

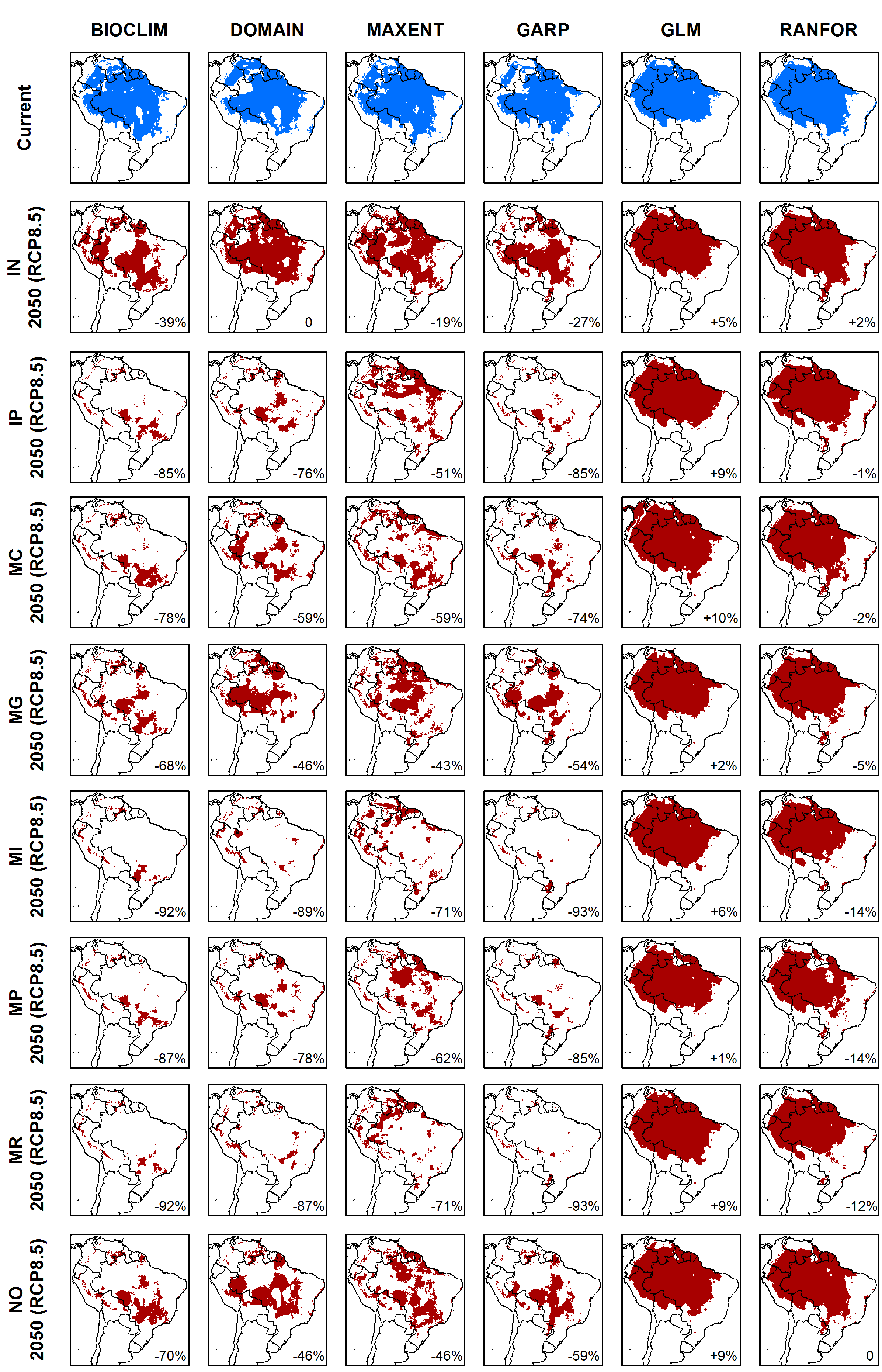

Supplement: S9 Fig — Each map shows binary model outputs. Future projections include the percentage of area lost or gain in comparison with current predictions. For names of each General Circulation Model, see S2 Table. (TIF) [file pone.0143282.s009.tif]
